# Correction: Kaposi’s sarcoma–associated herpesvirus stably clusters its genomes across generations to maintain itself extrachromosomally

**DOI:** 10.1083/JCB.20170201308082018c

**Published:** 2018-10-01

**Authors:** Ya-Fang Chiu, Arthur U. Sugden, Kathryn Fox, Mitchell Hayes, Bill Sugden

Vol. 216, No. 9, September 4, 2017. 10.1083/jcb.201702013.

The authors noticed that the cartoons in Figs. 3 and 8 lacked some of the intensity labels of the signals shown in the images below. While this oversight does not affect any conclusions, nor does it affect the raw data shown in the images of the cells in the figures, the authors wish to add the missing labels to the cartoons in Fig. 3, B1 and B2, and Fig. 8 A for the sake of completion.

**Figure 3.**
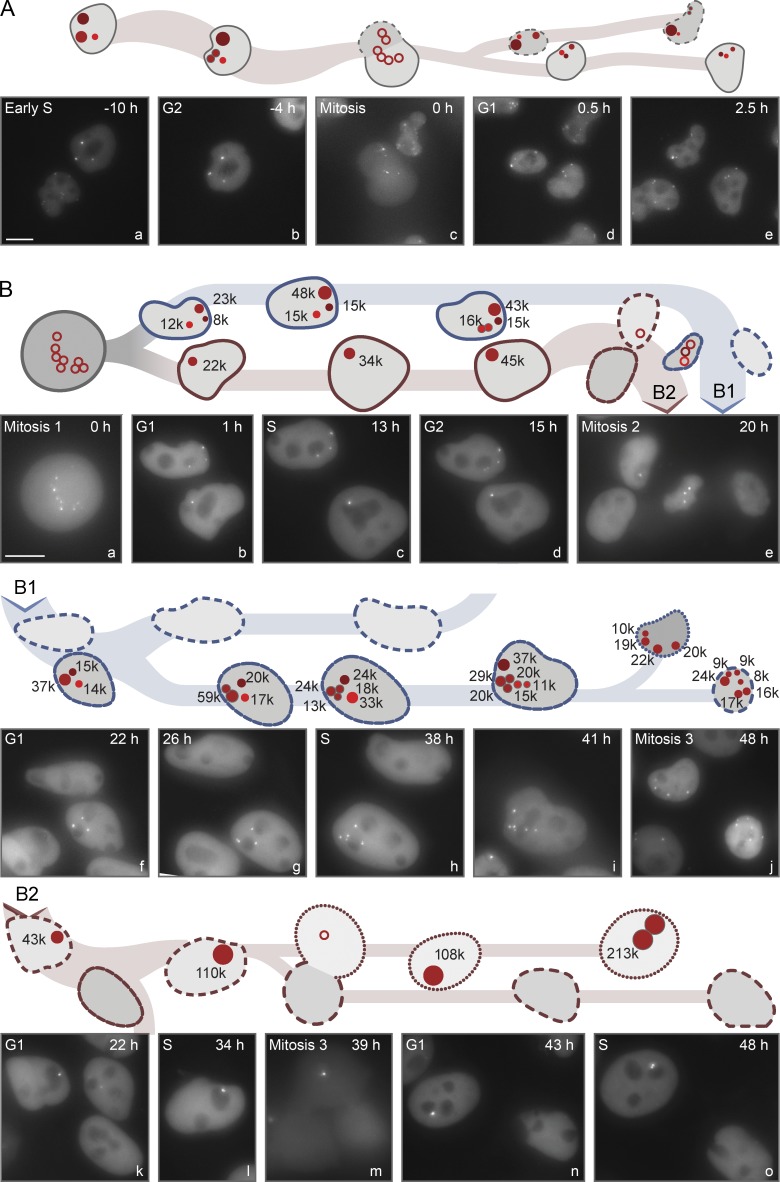
**Time-lapse analysis of KLacO and miniKLacO in live cells.** (A) Live-cell images illustrating clusters of KLacO plasmids and their segregation during mitosis. KLacO signals in SLK cells expressing the LacI-tdTomato fusion were followed over a 60-h period for at least two generations. Cartoons above the images depict the imaged cells with their intensities (a.u.) approximated by the diameters of the signals in the cartoons. Intensities cannot be measured reliably in mitosis, so these signals are unfilled in the cartoon. A given cell and its progeny are identified by having related outlines. A representative example is shown in which images from multiple z-planes are compressed: signals in the parental cell (a) differ in their intensities; one signal dissociates into two signals (b) with lower intensities before mitosis (c); each daughter cell receives three KLacO signals, with different intensities (d); one signal dissociates in G1 (e), illustrating that clusters of KSHV plasmids can dissociate throughout the cell cycle. (B) Series of images illustrating the growth of clusters of miniKLacO and their segregation in SLK cells expressing LacI-tdTomato and LANA1 during three sequential mitoses. All signals increased in their intensities (given in the cartoons) close to twofold each S phase, as measured in CAPS (c, h, and l). The varying intensities are readily observed in Video 1 (part A), which shows consecutive uncompressed *z*-planes for several of the compressed images in this figure. Bars, 10 µm. See also Fig. S1 and Tables S1 and S2.

**Figure 8.**
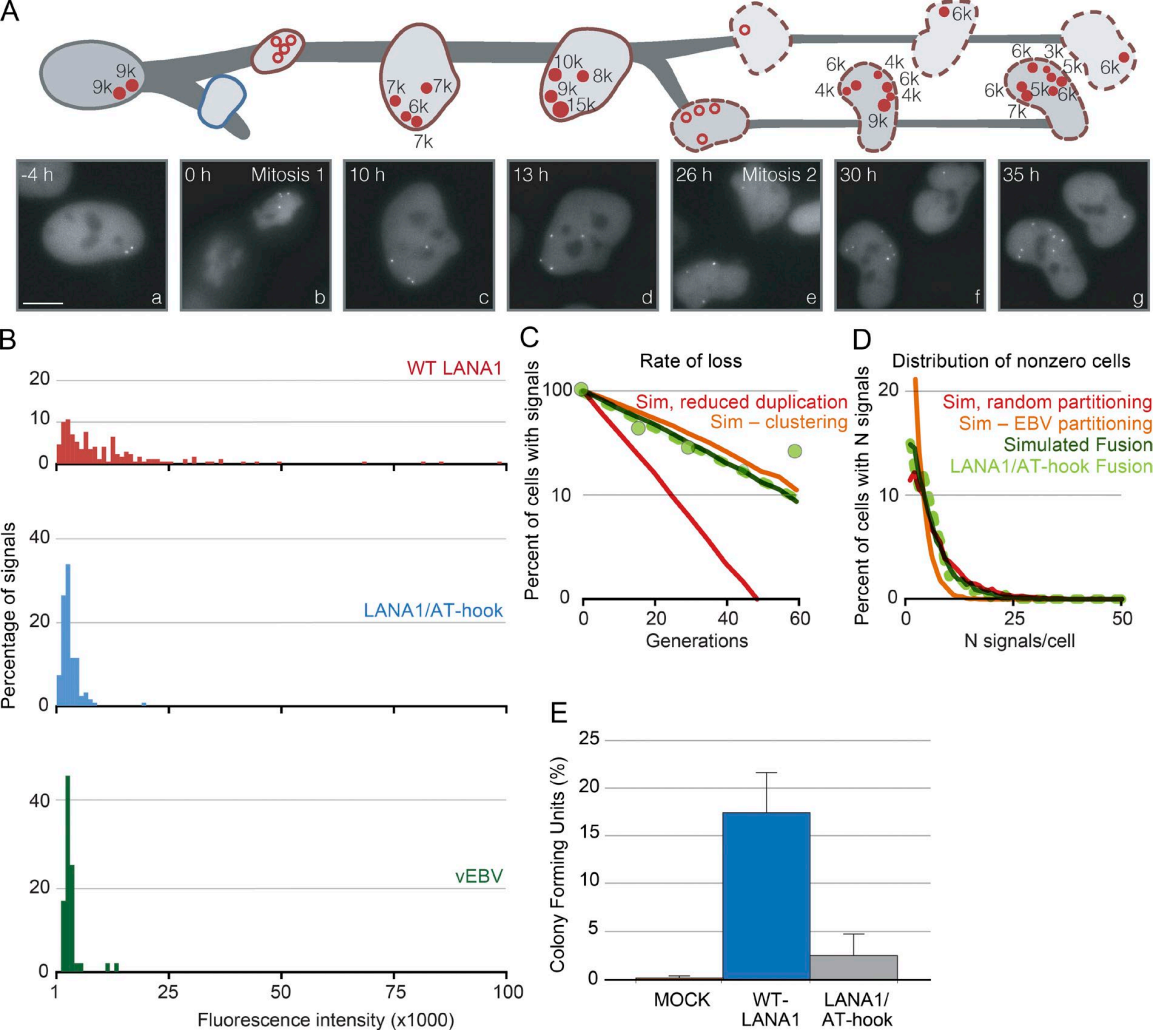
**Cluster formation of miniKLacO requires the N terminus of LANA and fosters plasmid establishment.** (A) MiniKLacO in SLK cells expressing LANA1/AT-hook was followed over 40 h using live-cell imaging. The cartoon and images with compressed signals are represented as described in the legend to Fig. 3. The varying intensities are readily observed in Video 1 (part B), which shows each consecutive *z*-plane for several of the compressed images in this figure. Bars, 10 µm. (B) Intensities of miniKLacO established in live SLK cells expressing wild-type LANA1 (red) or LANA1/AT-hook (blue), and those of visible EBV (green) in 293 cells were determined 3–4 h after mitosis (in G1 phase) with CAPS. They show that miniKLacO signals maintained by LANA1 are heterogeneous, with 100-fold differences in their intensities, whereas those maintained by LANA1/AT-hook or visible EBV are homogeneous, with two- to fourfold differences in intensities (wild-type LANA1, *n* = 169 signals; LANA1/AT-hook, *n* = 121 signals; vEBV, *n* = 46 signals). (C) Computer simulations predicted that the rate of loss of miniKLacO over time in cells expressing LANA1/AT-hook was insensitive to the absence of clustering but not to the rate of synthesis. (D) Two clones of SLK/LacI-tdTomato cells expressing LANA1/AT-hook and carrying miniKLacO were cultured without selection for 50 generations, and the distributions of miniKLacO signals were determined by FISH. The distributions of miniKLacO signals remaining in cells determined by FISH (light green line) were simulated (dotted blue line). A simulation using either the quasi-faithful partitioning of EBV (orange) or one using random partitioning (red) could not accurately reproduce the measured distributions. (E) WT LANA1 enhances colony formation relative to a derivative lacking its N terminus. SLK cells were transfected with a mock plasmid or plasmids expressing either wild-type LANA1 or LANA1/AT-hook along with miniKLacO and subsequently plated into selective media for 20 d to measure their colony-forming ability. The colony-forming unit was determined as a percentage of transfected cells resulting in puromycin-resistant colonies; error bars represent SDs obtained from three independent experiments. See also Fig. 7 and Table S3.

Both the HTML and PDF versions of the article have been corrected. These errors appear only in print and PDF versions downloaded on or before August 21, 2018.

